# Ileoileocolic Type Intussusception With Double Enhanced Layer Sign in a Pediatric Patient: A Case Report

**DOI:** 10.1002/ccr3.73707

**Published:** 2026-10-08

**Authors:** Naoya Sakamoto, Yuuki Take, Yuri Nemoto, Kentaro Ono, Yasunari Tanaka, Kouji Masumoto

**Affiliations:** ^1^ Departments of Pediatric Surgery Institute of Medicine, University of Tsukuba Tsukuba Ibaraki Japan

**Keywords:** computed tomography, double‐enhanced layer sign, ileoileocolic‐type, intussusception, pediatric surgery

## Abstract

Ileoileocolic intussusception can present without classic ultrasonographic target signs. A hyperattenuated inner bowel wall creating a “double‐enhanced layer” on contrast‐enhanced computed tomography (CT) may mimic viable bowel despite transmural necrosis and represents a diagnostic pitfall requiring early surgical intervention.

## Graphical abstract

The apparent double–layered enhancement represents hemorrhage within the necrotic inner layer, causing high attenuation, while the outer layer shows true contrast enhancement of the viable bowel wall.

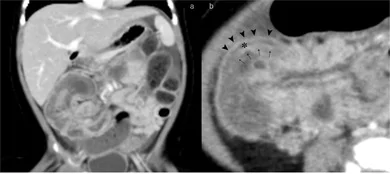

## 1 Introduction

Intussusception is a common cause of acute abdomen in children [1]. However, diagnosis can be particularly challenging due to atypical imaging findings and the absence of classic ultrasonographic features in cases of ileoileocolic‐type intussusception [2]. Although the “target sign” is a well‐known ultrasonographic finding indicative of intussusception [3], we encountered a 4‐month‐old infant who did not exhibit this typical feature. Here, we report the diagnostic pitfalls encountered in this case and highlight the imaging characteristics of ileoileocolic‐type intussusception.

## 2 Case History

A 4‐month‐old boy presented to another hospital with vomiting the previous day. There was no history of diarrhea or loose stools, and the clinical course did not suggest enteritis. The vomit was green and had a bile‐like appearance. A rectal examination could not be performed due to the patient's small size. Vital signs were as follows: temperature 37.4°C, pulse 124 bpm, blood pressure 79/48 mmHg, and respiratory rate 41 breaths per minute.

Peripheral oxygen saturation (SpO_2_) was 100% (room air), and no deterioration in oxygenation was observed. Physical examination revealed slightly decreased vitality. The abdomen was distended, and bowel sounds were diminished. However, it was unusual that there were almost no signs of peritoneal irritation suggestive of peritonitis. Massive hematochezia was observed, laboratory investigations revealed early‐stage inflammation (white blood cell count [WBC] 15,900/μ, C‐reactive protein [CRP] level of 3.84 mg/dL) and slight anemia (hemoglobin [Hb 12.4] g/dL). Arterial blood gas analysis showed a pH of 7.48; however, the HCO_3_^−^ level was 19.3 mmol/L, base excess at −2.6, and lactate at 1.0. These findings suggested compensated metabolic acidosis. Abdominal radiography demonstrated findings consistent with ileus; therefore, contrast‐enhanced computed tomography (CT) was performed, which revealed a closed‐loop configuration. Based on the diagnosis of strangulated ileus, the patient was emergently transferred to our hospital. The CT scan images revealed bowel within bowel appearance, which had not been recognized preoperatively. Retrospective review of the CT images revealed a characteristic double‐enhanced layer appearance (Figure 1).

**FIGURE 1:**
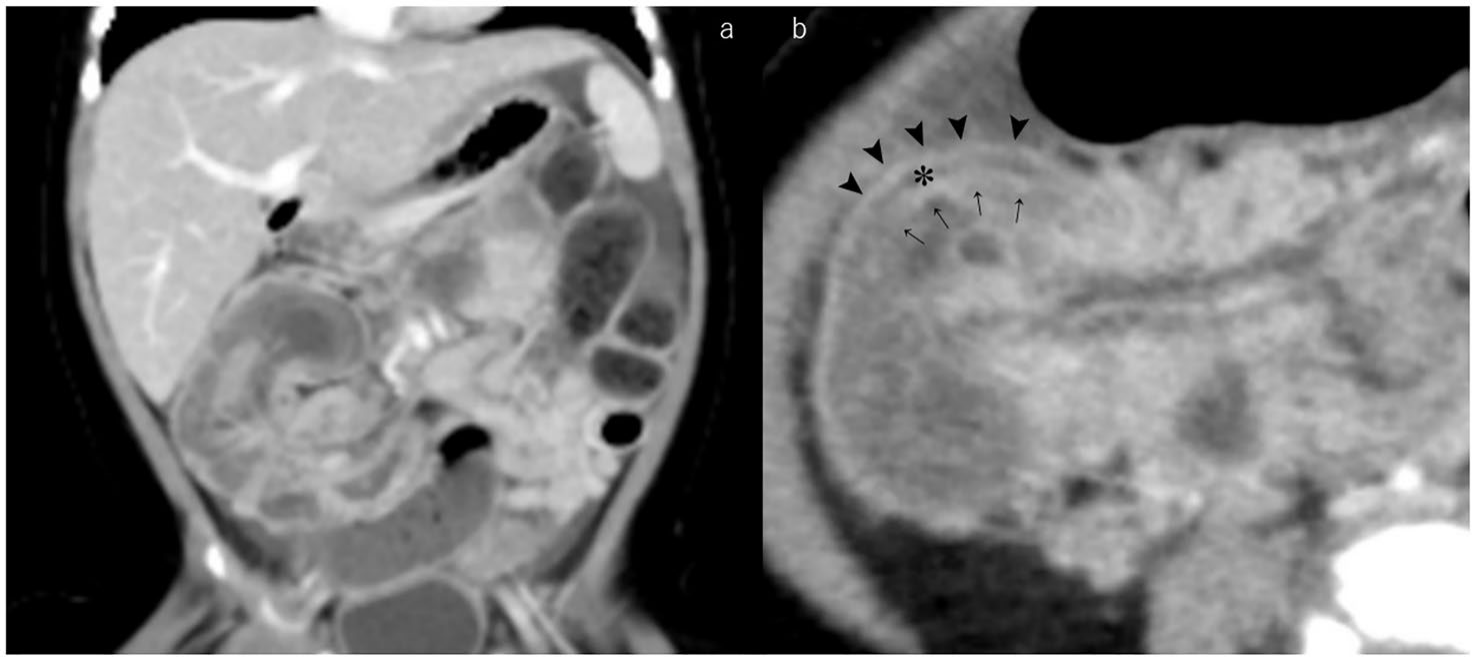
Contrast‐enhanced CT images showing frond‐like loops of small bowel (a). (b) Retrospectively, the inner layer (arrow) represented necrotic intestine, whereas the outer layer (arrowhead) corresponded to viable colon with preserved blood flow. Fluid accumulation is also noted (✽). The inner layer appears discontinuous.

The characteristic “target sign” of intussusception was not observed, and only an absence of blood flow (Figure 2) was identified. In retrospect, these findings were the result of hemorrhagic and necrotic tissues.

**FIGURE 2:**
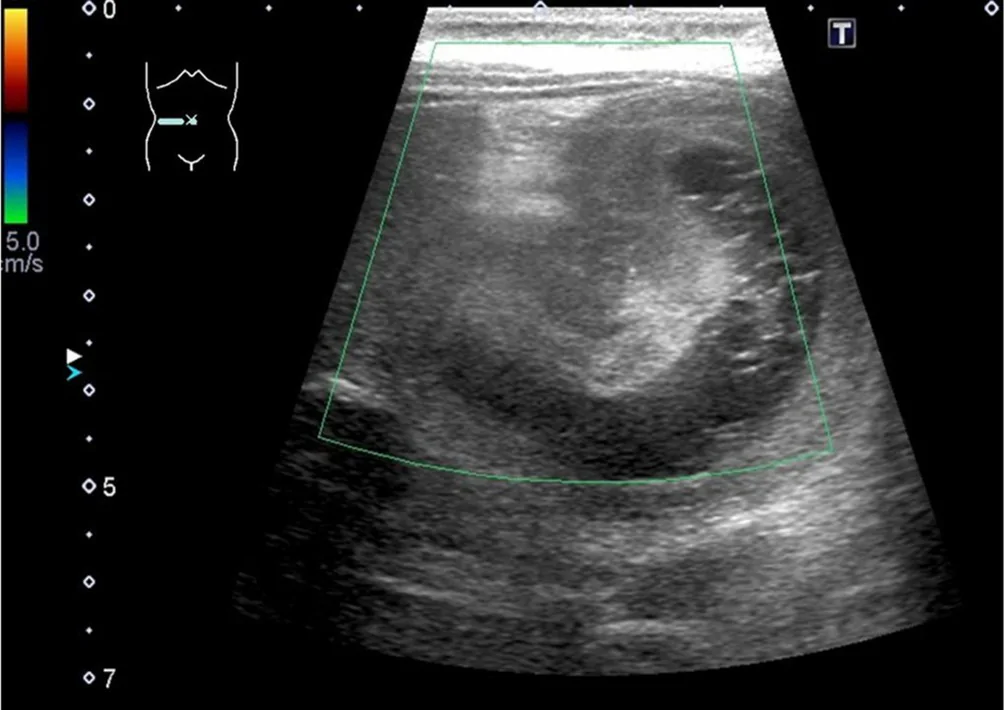
Color Doppler ultrasonography showing no detectable blood flow within the intestinal wall. Despite the contrast‐enhanced CT demonstrating a double‐enhanced layer, perfusion was absent on Doppler imaging.

## 3 Differential Diagnosis, Investigations, and Treatment

Although intussusception was suspected, a definitive diagnosis could not be established before surgery. In Japan, the rotavirus vaccine is administered within 14 weeks of birth. The patient had received rotavirus vaccination; however, more than 1 month had passed since the patient's vaccination, so it was unlikely that the symptoms were caused by a side effect of the rotavirus vaccine. Differential diagnoses included internal hernia and strangulated ileus caused by a Meckel's band. Therefore, we proceeded with emergency surgery. A nasogastric tube was not inserted preoperatively because the patient was immediately transferred to the operating room. By the time the nasogastric tube was inserted in the operating room, the patient had already vomited, and the amount of contents was small.

Upon intra‐abdominal inspection, the necrotic bowel was not identified. Careful palpation of the bowel revealed a firm mass within the colon. At that point, an intraluminal colonic lesion was suspected for the first time, and a colotomy was performed. Necrotic bowel was identified within the colonic lumen (Figure 3). Based on these findings, the diagnosis of intussusception was confirmed. Because manual reduction using Hutchinson's maneuver was unsuccessful, a 30‐cm section of the small intestine, including the necrotic portion, was resected. The colon was not resected, and the ileocecal valve was preserved. A double‐barrel ileostomy was created. The anal‐side small bowel was brought out as a mucous fistula. Two months after the initial surgery, stoma closure was performed. At the 1‐year follow‐up, the patient was tolerating oral feeding without difficulty and demonstrated appropriate physical growth. There was no recurrence of intussusception, and abdominal radiography revealed no evidence of intestinal obstruction.

**FIGURE 3:**
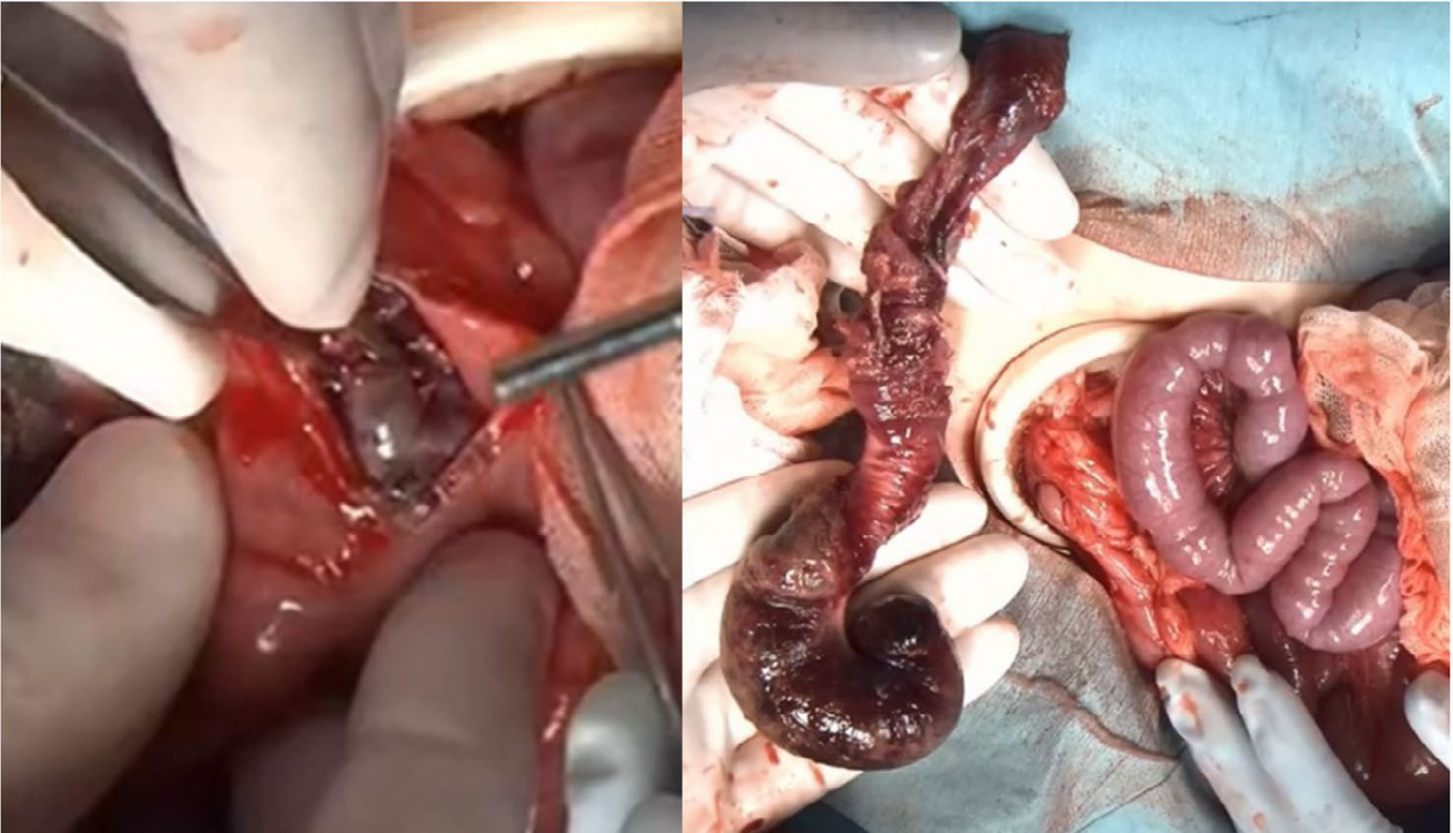
Intraoperative findings. The necrotic small intestine was identified within the viable colon. The right panel shows the entire resected necrotic segment.

## 4 Conclusion and Results

On examination of the resected specimen, the ileum was found to be invaginated into the colon, confirming the diagnosis of ileoileocolic‐type intussusception (Figure 4). The mesenteric vessels and fat within the invaginated segment were completely necrotic and liquefied. The patient recovered uneventfully after ileostomy closure and was discharged without complications. Because the necrotic bowel was located within the normal bowel, identification of the necrotic segment was time‐consuming. If this had been anticipated preoperatively, the operative time could have been reduced and the burden on the patient minimized.

**FIGURE 4:**
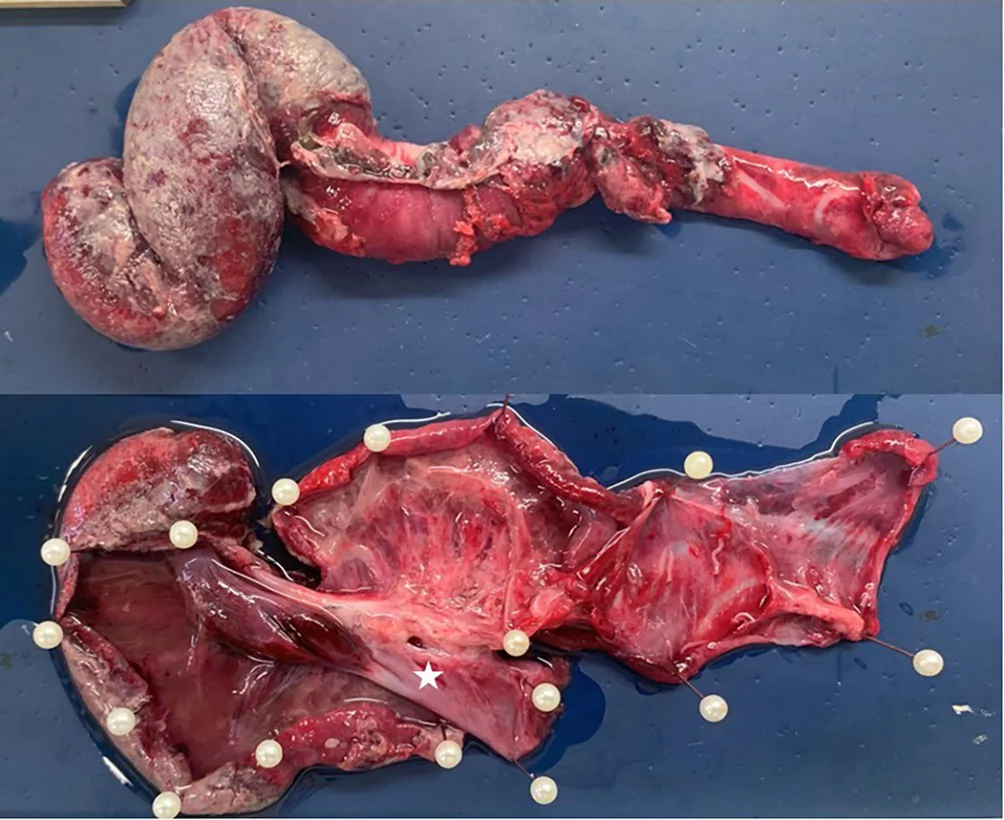
Pathological findings. The ileum (★) was invaginated into the colon, confirming the diagnosis of ileoileocolic‐type intussusception. Note the complete necrosis and liquefaction of the mesenteric fat and vessels within the invaginated segment.

## 5 Discussion

The most common type of intussusception is the ileocolic type [1]. Ileocolic intussusception can be easily diagnosed based on ultrasound findings, whereas the ileoileocolic type is rare and often more difficult to diagnose [1, 2]. This type is associated with a significantly higher incidence of bowel necrosis [4]. The presence of bloody stool is a clinical finding that strongly suggests bowel necrosis [5]. Although inflammation secondary to bowel necrosis generally leads to peritoneal signs, in intussusception, exudate from the necrotic segment is released into the colonic lumen, potentially accounting for the lack of peritoneal irritation observed in the present case. Although more than 90% of intussusception cases in children are idiopathic, approximately 5% are attributable to a pathological lead point, including lymphoid hyperplasia, Meckel's diverticulum, duplication cysts, intestinal polyps, mesenteric lymphadenopathy, lymphoma, prior surgery, or trauma [5, 6]. In this case, it was not possible to determine the cause of the intussusception. Furthermore, due to the small number of cases, it was not possible to identify any region‐specific causes. The success rate for reducing ileoileocolic intussusception is only 10% [2]. Therefore, ileoileocolic intussusception should be diagnosed promptly, and surgical intervention rather than reduction by high‐pressure enema should be performed.

Ultrasonography is the first‐line diagnostic modality in children. The characteristic “target” and “doughnut” signs were first described in 1985 [3], and ultrasonography currently has a reported sensitivity of approximately 95% [7]. However, when ultrasound findings are atypical, CT should be considered [8]. In our case, the classic target sign was absent on ultrasound. This can be explained by the following pathological findings: (1) necrosis and thickening of the incarcerated bowel, (2) necrosis and dissolution of the mesenteric fat and vessels, and (3) distortion of the bowel configuration due to necrosis of the mesentery.

Hogan et al. reported that contrast enema examination demonstrated a multipolypoid appearance [9]. Peh et al. reported that these findings appeared as frond‐like loops on ultrasonography [2]. In our case, contrast‐enhanced CT indicated that a multipolypoid and frond‐like loops appearance represented extensive small bowel invagination, with the necrotic segment being surrounded by viable bowel wall. On contrast‐enhanced CT, the bowel wall, which was presumed to be necrotic, exhibited hyperattenuation and appeared similar to viable bowel. The hyperattenuation is thought to be attributable to capillary hemorrhage originating from the necrotic bowel wall [10]. This imaging appearance may lead to an underestimation of bowel necrosis and potentially delay appropriate surgical intervention. We initially interpreted the inner wall as viable tissue; however, it actually represented necrotic mucosa with hemorrhage. It is crucial not to misinterpret this hyper attenuated inner layer as viable intestine. Recognition of this finding may provide a key clue in diagnosing ileoileocolic‐type intussusception.

To our knowledge, this is the first report describing a double‐enhanced layer appearance on contrast‐enhanced CT in ileoileocolic intussusception mimicking viable bowel.

## Author Contributions

**Naoya Sakamoto:** conceptualization, data curation, writing – original draft, writing – review and editing. **Yuuki Take:** investigation, writing – review and editing. **Yuri Nemoto:** data curation, writing – review and editing. **Kentaro Ono:** conceptualization, writing – review and editing. **Yasunari Tanaka:** conceptualization, writing – review and editing. **Kouji Masumoto:** project administration, writing – review and editing.

## Funding

The authors have nothing to report.

## Consent

Written informed consent for publication of this case report and any accompanying images was obtained from the patient's parents.

## Conflicts of Interest

The authors declare no conflicts of interest.

## Data Availability

Data sharing was not applicable to this article as no datasets were generated or analyzed during the current study.
